# The ecology of ageing in wild societies: linking age structure and social behaviour

**DOI:** 10.1098/rstb.2022.0464

**Published:** 2024-10-28

**Authors:** Joe P. Woodman, Samin Gokcekus, Kristina B. Beck, Jonathan P. Green, Dan H. Nussey, Josh A. Firth

**Affiliations:** ^1^ Edward Grey Institute of Field Ornithology, Department of Biology, University of Oxford, Oxford OX1 3SZ, UK; ^2^ Senckenberg Biodiversity and Climate Research Centre, Frankfurt am Main, Germany; ^3^ Institute of Ecology & Evolution, The University of Edinburgh, Edinburgh EH9 3JT, UK; ^4^ School of Biology, University of Leeds, Leeds, UK

**Keywords:** ageing, age structure, senescence, sociality, social behaviour, social structure

## Abstract

The age of individuals has consequences not only for their fitness and behaviour but also for the functioning of the groups they form. Because social behaviour often changes with age, population age structure is expected to shape the social organization, the social environments individuals experience and the operation of social processes within populations. Although research has explored changes in individual social behaviour with age, particularly in controlled settings, there is limited understanding of how age structure governs sociality in wild populations. Here, we synthesize previous research into age-related effects on social processes in natural populations, and discuss the links between age structure, sociality and ecology, specifically focusing on how population age structure might influence social structure and functioning. We highlight the potential for using empirical data from natural populations in combination with social network approaches to uncover pathways linking individual social ageing, population age structure and societal functioning. We discuss the broader implications of these insights for understanding the social impacts of anthropogenic effects on animal population demography and for building a deeper understanding of societal ageing in general.

This article is part of the discussion meeting issue ‘Understanding age and society using natural populations’.

## 1. Introduction

Age determines many aspects of life, underpinning variation in individual-level characteristics across species [1–4]. This is summarized through the framework of life-history theory, which posits that organisms have limited resources invested in traits and processes at different points throughout their lifespan to maximize fitness [5]. Ageing in late life is generally associated with senescence, i.e. a decline in physiological functioning that leads to a loss of organismal function, decreased fecundity and increased probability of death [6–13]. However, ageing itself broadly reflects a temporal parameter that measures the amount of time since birth, and therefore, may be accompanied by many other changes in an individual’s biology in addition to physiological senescence in late life, such as sexual maturation, the accumulation of resources and social experience, or a changing social environment due to cohort effects and selective disappearance resulting from natural selection acting within a generation. Therefore, patterns of age-specificity in individual characteristics can be complex but are evidenced in reproduction and survival probability [14–20], physiology and morphology [21–25] and behaviour [26–35]. Much previous research has studied ageing in laboratory settings, particularly using insects and other short-lived animals as models [9,10,36–38]. However, studies on captive animals may lead to conclusions that cannot be generalized to natural ecological contexts [39]. Therefore, the importance of studying ageing in wild populations is widely acknowledged [18,40–45].

An individual’s age can have consequences not only for its own survival and behaviour but also for the functioning of the population of which it is part. Recent work highlights that individual social behaviour can change with age [26–35,46–48], for example in terms of how many associates an individual has. This might be driven by a number of mechanisms [34] such as age-related changes in experience [49–52], space-use [26], cognitive physiology [53–55] or phenotypic plasticity [56,57]. Much of the research that has assessed age-related differences in sociality does so through comparing individual social behaviour among different age classes, as opposed to using longitudinal studies which measure how ageing relates to changing sociality within individuals across their lifetime. Thus, age-related differences in social behaviour may not be a direct result of within-individual ageing, but also between-individual processes such as cohort effects or selective disappearance [58–60]. Crucially, where age relates to social behaviour through whichever of the discussed mechanisms, and thus variation in the number, type and strength of relationships formed, the age profile of the population as a whole might be expected to influence the overall social organization and functioning, and the consequences that depend on this. This can be conceptualized using the perspective of social structure, which is a synthesis of all social relationships between members of a group. It is determined by social interactions among individuals, from which relationships form, and thus govern the overall social structure of a group or population [61,62]. Hence, though frequently overlooked, the age structure is thus likely to be an important driver of variation in social structure across populations [45].

**Figure 1.**
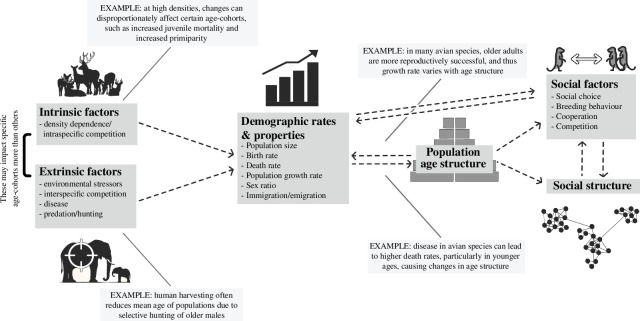
A conceptual synthesis of how variation in demographic rates and properties, age structure and sociality might mutually affect one another.

Age structure is a demographic property that describes the distribution of age within a population, determined by variation in processes that affect how many individuals are born, die and migrate in and out of a population. It is well established that the variation in age structure plays an important role in the demographic functioning of populations. This is because individual age-specificity in survival and reproduction means that fluctuations in age structure influence population vital rates [63,64]. Additionally, age groups differ in their demographic sensitivity to density dependence and environmental factors [65–68]. Thus, variation in age structure influences the overall population growth rate, which itself will cause a change to age structure as more or fewer individuals are recruited into the population or die [69–75]. Therefore, age structure and the demographic processes that determine it are highly interrelated and exert a reciprocal influence on one another (figure 1). As already explained, however, age structure will not only influence demographic rates but may also affect the social structure of populations and the operation of social processes within them. The interplay between age and society is of primary significance in a range of biological disciplinaries: to behavioural ecologists interested in the causes and consequences of social processes, and how this is shaped by age [31,58,76–80]; to evolutionary biologists concerned with the evolution of social behaviour and ageing, and how evolution influences social structure over generations [1,8,16,81–84] and to gerontologists interested in ageing human societies [85–88]. However, our general understanding of how population age structure affects sociality in the wild is limited.

In this article, we assess previous research into age-related effects on social processes with the aim to better understand the link between age structure and sociality in the wild (§2). While it is clear that age structure, sociality and the ageing process can profoundly influence the evolutionary dynamics of each other [3,89–96], this review is primarily focused on the ecological perspective of the link between age and sociality in wild populations. Finally, we highlight the potential for using empirical data from natural populations in concert with a social network approach to uncover the causes and consequences of the relationship between age structure and sociality and discuss future directions for the research field (§3).

## 2. Population age structure and sociality

Existing work on natural populations identifies the potential for age structure and demographic rates to be tied to one another in various ways (figure 1). For example, for many European bird species, variation in masting events (e.g. in beech *Fagus*) affects juvenile survival and recruitment [97,98]. As a consequence, considerable temporal variation in age structure is generated: in great tits (*Parus major*), for example, the proportion of the population consisting of yearlings can vary from 27 to 68% [99]. Age-specificity in reproduction and response to density dependence in this species [100–105] means that such changes in age structure will affect the population growth rate. What remains to be understood is the role sociality plays in the determination of age structure and demographic rates in natural populations.

The role that sociality plays in affecting variation in population age structure is currently not well understood but may be significant. This is because the patterning of social relationships, which produce overall social structure, can mediate survival and reproduction, thus influencing birth and death rates and the resulting distribution of age in wild populations [106]. For example, foals with a higher number of associates in a feral horse (*Equus caballus*) population had greater survival following a catastrophic event that caused a loss of 40% of individuals [107]. Benefits to health and survival as a result of social cohesion have also been evidenced in killer whales (*Orcinus orca* [108]); giraffes (*Giraffa camelopardalis* [109]); bighorn sheep (*Ovis canadensis* [110]); rock hyrax (*Procavia capensis* [111]), yellow-bellied marmots (*Marmota flaviventris* [112,113]), Barbary macaques (*Macaca sylvanus* [114,115]), rhesus macaques (*Macaca mulatta* [116–118]); baboons (*Papio cynocephalus* [119–121]) and humans (*Homo sapiens* [122–125]). Indeed, such benefits may help to explain why individuals increase their social connections after major disturbances [126–128]. Conversely, in some contexts, increased sociality may reduce survival or reproduction [124,129–132], for instance, when social contact increases infection risk [133–138]. In these ways, social behaviour might directly influence vital rates and generate variation in the resulting age structure of wild populations.

While the social behaviour and resulting social structure of a population may influence its age structure, we can also conversely ask whether age structure might affect the social structure and functioning of social processes. Such social processes refer to behavioural interactions including two or more individuals, affected by age-specific tendencies to perform them, and the overall structure of the social network. Age-specific social behaviour has been demonstrated in many animal taxa from laboratory, domestic and wild populations [26–35]. In some cases, changes in social behaviour with age are profound. For example, as male lions (*Panthera leo*) age, they move from their natal pride into coalitions with other older males [139], thus considerably altering their social associations. Therefore, age structure might be critical to the overall social structure of wild populations. Some research has considered age distribution in social networks, asking in particular whether groups exhibit assortment with respect to age. Age-assortment in social networks, whereby association between same- or similar-age individuals is stronger than that expected from chance, has been observed in birds [29,140,141], primates [142] (including humans [143]), yellow-bellied marmots [80], sea lions (*Zalophus wollebaeki* [144]) and potentially bottlenose dolphins [145]. Such age-assortment may interact with the influence of age on social behaviour at the individual level to provide a mechanism whereby the overall age structure influences the emergent social structure and the operation of social processes within the social network. Despite this, the causal effect of age structure on the functioning of social processes is relatively understudied, and few studies have explicitly considered the mechanisms through which age structure determines social behaviour and structuring in wild populations. Here, we explore this by assessing how age is known to affect the relationship that population age structure holds with four key social processes: (i) social choice; (ii) breeding behaviour; (iii) cooperation; and (iv) competition.

### (a) Social choice

Social preferences and relationships can influence survival and life-history outcomes in social species [95–105,107–112], as the choices made in terms of who to associate with and for how long can influence success in various contexts such as mating, cooperation, competition and social learning. It is well established that physiological characteristics can change with age, and senescence in such traits with old age is a widespread phenomenon [8,18]. The neurological and hormonal mechanisms that underpin social choice have been studied extensively in laboratory settings [146–150]. For example, the neuropeptide oxytocin is particularly important in mediating social choice in humans, such as that involved in parent–offspring bonds [151,152]. However, senescence in the physiological properties that underpin social behaviour and its relation to social changes associated with ageing in wild populations is understudied, and we lack a general cross-species understanding of patterns of social senescence (see §3).

In the context of social choice, humans become more selective with age, as individuals invest in fewer but stronger relationships [153–156]. Evidence is now emerging for similar patterns of social selectivity with increased age in non-human animals including: chimpanzees (*Pan troglodytes* [33]), macaques [32,157–159], yellow-bellied marmots [59,80], red deer (*Cervus elaphus* [26]), killer whales [160] and common terns (*Sterna hirundo*) [47]. In marmots, for example, fewer attempts are made to interact with old individuals, which consequently exert less social influence [59]. Observed patterns of increasing social selectivity with age might emerge from different, and potentially simpler processes, in wild populations compared to human societies, for example through increased mortality of older social partners or changes in space-use and associated social interactions. For example, in red deer, older individuals are less socially connected which may stem from changes in space-use, with older deer having smaller home ranges in areas of lower quality and density [26].

It is likely that these age-related changes in social choice will play a role in the relationship between population age structure and other social processes. For example, if strong mutual bonds between older individuals promote prosocial behaviour, the presence of older individuals within a population may contribute to higher average rates of cooperation. Overall, age-related changes in social choice may influence social structure through changing which, and with how many, associates an individual chooses to interact with depending on age. This, therefore, provides a mechanism by which variation in age structure may affect the overall societal structure and functioning.

### (b) Breeding behaviour

Breeding behaviour is a well-studied aspect of social behaviour, and age-related changes might mean that variation in age structure will alter patterns of breeding across a population. Here, we review the implications of age-specificity for breeding processes that depend on social interactions, through mate choice and subsequent decisions of whether to form a long-term partner social bond, divorce or commit extra-pair copulations (EPCs). We assess how these might affect population-level breeding behaviour given variation in age structure.

The choice of mate can be an important determinant of reproductive success [161–167]. It has been demonstrated that some females adjust mating preferences based on previous experience, known as the ‘previous male effect’ [165–178]. Because refinement of mating preferences occurs in response to previous mating behaviour, as older females will have undergone more breeding attempts, they may then be expected to show more refined mating preferences than younger females. This effect captures how age relates to mate choice, since females with greater experience must have undergone more breeding attempts, and therefore older individuals may be better at choosing mates [49]. Conversely, in some cases, older females might be less choosy, which may be caused by delayed mating in senescent females resulting in reduced choosiness, or decreased ability to discriminate male quality due to deterioration of sensory capacity with senescence [179–181]. As well as previous experience, mate preferences can be learnt socially, a process known as mate copying [182–186]. In some species, younger individuals are more likely to copy the mate choice of others [187–189], and thus age structure might influence the overall levels of mate copying, which could have considerable effects on population-level breeding behaviour through affecting which males are chosen. Furthermore, as well as influencing population-level breeding behaviour through individual age-specificity, population age structure might mediate mate choice by determining which individuals of a given age mate together if the age distribution is skewed towards specific age-cohorts. For example, recent work demonstrates that in species with high mortality rates, a large proportion of the population exists in a single age-cohort, and thus fluctuations in age structure largely determine variation in levels of age-assortative mating [99,190].

In socially monogamous species, once a mate is chosen, individuals may remate with the same partner to increase breeding success [191–193]. Such remating results in pair-bonding behaviour, where a long-term relationship forms [194–197]. Pair-bonds require that partners sustain their relationship beyond a single or multiple mating attempts [194,198,199] and when individuals elect to remate based on previous success [200,201], we may expect to see a higher proportion of older individuals pair-bonded than younger ones, due to age-specific breeding success in many species where performance is lower in young breeders [15,19,202]. Age structure might therefore influence pair-bonding in populations, which may have important consequences as pair-bonding can be adaptive independent of age and reproductive experience [203], thus potentially affecting population productivity. However, this relationship is complicated by the fact that, as pairs age, there is an increasing likelihood that one partner will die between breeding attempts, leading to widowing [192,204]. Moreover, in short-lived species where mortality between breeding attempts is high, costs of waiting to remate with a partner that has died have been hypothesized to select for divorce and partner-switching [205]. The strength and direction of the relationship between individual age and pair-bonding behaviour are thus likely to be mediated by mortality and lifespan, with the prediction that population age structure should most strongly predict pair-bonding across populations in long-lived species with low extrinsic mortality.

In addition to avoiding costs associated with delayed breeding, an individual may divorce if it fails to reach optimum reproductive potential with a partner of low quality [204–207]. Within a population, the proportion of prime-age individuals (those in the age class with the highest reproductive and survival rates [65,208–210]) may affect divorce rates, as partners choose to divorce to mate with individuals of higher reproductive value. For example, divorce rates in barnacle geese (*Branta leucopsis*) increase when there is a greater proportion of older, more experienced individuals among unpaired birds [191,192]. In some cases, rather than divorcing their partner, individuals may seek EPCs [211,212]. The likelihood of performing EPCs can be influenced by age, with meta-analyses pointing to a positive correlation between male age and extra-pair paternity gained from EPCs [213,214]. Thus, population age structure is likely to influence rates of both divorce and EPCs, which may in turn have a significant influence on population-level breeding behaviour depending on the distribution of age within the population.

### (c) Cooperation

There is emerging evidence for a close relationship between age and cooperation across multiple ecological contexts, and in some cases, there is a clear association between age structure and population-level measures of cooperation. For example, a study of 16 populations in a small-scale horticulturalist human society has demonstrated that demographic factors influence resource-sharing [215]. Age, in particular, had a positive effect on resource-sharing, with older individuals contributing more to the ‘group pot’. Furthermore, villages with more adult sisters had higher inequality in resource distribution, suggesting an interplay between age structure, sex distribution and kinship in explaining rates of cooperation. Some empirical evidence also demonstrates ecological links between cooperation and age in non-human animals [216–218] and bacteria [219], but the influence of variation in population age structure has not been explicitly considered.

Levels of tolerance and willingness to cooperate may be expected to vary over an individual’s lifespan, related to changes in pay-offs, partner-choice, competitiveness and the learning of heuristics that allow individuals to benefit from cooperative interactions. Older individuals may have more familiar associates and stronger bonds, allowing for more frequent cooperation with their social associates. For example, great tits are more likely to cooperate with familiar neighbours [220], and older individuals are more likely to be familiar with their neighbours [221]. Therefore, in such cases, populations with many older individuals may have higher rates of cooperation overall. Furthermore, cooperation may increase with age if individuals learn to cooperate through their experiences with other cooperators. However, as individuals age, the number of social partners may dwindle if partners are not replaced upon their death, potentially leading to lower levels of cooperation through loss of opportunity [26]. Alternatively, the number of social partners may be reduced due to the previously discussed potential increases in social selectivity with age. Even if age is not directly related to the propensity to cooperate, it is possible, for example, that if individuals of a particular age are more likely to engage in policing of cheaters, the age structure of the population may influence rates of cooperation versus defection [222]. Furthermore, if cooperation confers survival or reproductive benefits to cooperators, individuals may cooperate more as they age in order to mitigate the potential negative effects of senescence [220,223,224] (see §3).

An extreme form of cooperation seen in animals is cooperative breeding, where individuals provide care to young that are not their own (alloparental care). From an ecological perspective, cooperative breeding is considered to most commonly arise when individuals delay or forego natal dispersal and instead remain in their natal territory caring for the offspring of breeders [225]. In such systems, age-dependent plasticity in the provision of alloparental care may allow individuals to adjust their helping strategies to changes in social and environmental conditions that occur over their lifetime. Recent work shows that local relatedness to other group members can change systematically through the lifespan of an individual, known as kinship dynamics [83,218,226,227]. In cooperative breeders, relatedness between helpers and breeders commonly declines as helpers age, due to time-dependent breeder replacement and dispersal dynamics [218,228]. In these cases, individuals may reduce investment in help as they age [218,229], as lower relatedness often predicts decreased helping efforts in cooperative breeders [230–235]. In Damaraland mole-rats (*Fukomys damarensis*), for example, investment in alloparental care declines with age [236], although this effect may be due to more general age-related declines in performance. Moreover, a decline in relatedness with age, and with it the indirect fitness pay-offs of helping, might provoke dispersal attempts by older helpers which then seek to boost inclusive fitness through reproduction outside of the natal group [237]. In other species, however, the prospect of territory inheritance and associated reproductive benefits may favour continued philopatry. This occurs, for example in primitively eusocial hover wasps (*Liostenogaster flavolineata*), where females form an age-based queue in which only the oldest female reproduces [238–241]. In this and other species that queue for inheritance, individuals are observed to reduce investment in alloparental care as they ascend rank, which can be interpreted as an attempt by older and thus higher ranking individuals to reduce the mortality risk associated with foraging off the nest in an attempt to survive to inherit the nest [242]. Such a selfish strategy, therefore, leads to a similar negative relationship between age and helping effort, but in this case, the relationship is mediated by the prospect of direct fitness gains through future reproduction rather than the concurrent decline in relatedness and indirect fitness pay-offs of help. Multiple ecological processes can shape age-specificity in cooperative breeding, which may therefore in turn generate relationships between age structure and cooperation at the population level.

### (d) Competition

Competition for mates, breeding sites and food is a fundamental ecological process in wild populations [243], including in social species where individuals face local competition with group members. As with cooperative behaviours, an individual’s ability to perform, and investment in, competitive behaviours can be sensitive to age [244,245]. In some taxa, older individuals are dominant in competitive interactions [246–252], allowing them to monopolize resources [253]. Age too is observed to confer competitive dominance in species where males form reproductive alliances with the aim of monopolizing access to females. In bottlenose dolphins, for example, alliances comprising old males are more successful in competition against alliances of young males, despite typically comprising fewer individuals [254]. In some social species, costs of competition among group members favour the formation of dominance hierarchies, with differences in competitive ability reinforced through ritualized threat behaviours rather than escalated fighting [255,256]. Because competitive ability commonly increases with age, the age structure of populations can strongly influence the formation of hierarchies [257]. In *Polistes* wasps, for example, age structure is an important determinant of hierarchy formation due to an age-based system of queen replacement [258].

Variation in competitive ability with age will also have important consequences for density dependence in age-structured populations. The effect of age distribution on both inter- and intra-specific competition has been explored through the use of density-dependence models that mathematically estimate the outcomes of competition depending on age structure [65,68,259–263]. The use of such models alongside empirical data gives an indication of how age structure influences density dependence by mediating levels of competition. For example, in great tits, young individuals constitute the critical age class for density regulation, whereby the youngest birds have the strongest competitive effect on other breeding females of the same age or older [104]. Expanding these initial findings, it has been shown that including age-specific effects in density-dependence models improves the predictions of population size fluctuations by up to three times in a great and blue tit (*Cyanistes caeruleus*) population [105], indicating the importance of age structure in determining population-level competition.

Variation in age structure will also affect the probability that certain individuals win competitive encounters and which competitive strategies are adopted. For example, the competitive environment is strengthened in mixed-age *Plodia interpunctella* and *Ephestia cautella* moth cohorts compared to uniform-aged cohorts [264]. Furthermore, changes in age structure and the levels of competition might be mutually reinforcing in that competition may also lead to fluctuations in age structure through its effect on death or dispersal rates. For example, it has been shown that competition for breeding patches, mediated by the presence of predators, induces changes in age structure through age-specific dispersal away from the breeding site in Audouin’s gulls (*Ichthyaetus audouinii* [265]).

## 3. Future directions

We have sought to highlight the potential for variation in age structure to govern sociality in wild populations through its impact on social behaviour. However, the discussion of the effects of age structure on sociality at the population level is largely conjectural based on predictions from age dependence in behaviour mostly at the individual level. We argue that wild animal populations provide a unique opportunity to advance knowledge regarding the relationship between age structure and sociality as it manifests explicitly at the population level. This is because natural populations often show considerable variation in age composition across space and time in well-monitored systems; and also provide a useful setting for the fine-scale tracking of individuals over their entire lifetime and the monitoring of their social networks (and associated social processes) over many generations. In §3a–d, we discuss future emerging directions for this area.

### (a) Advancing social network approaches in relation to ageing in wild populations

**Figure 2.**
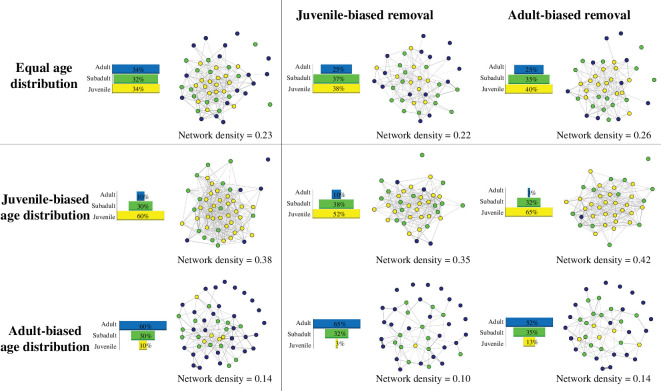
Social networks of hypothetical populations with different age structures following juvenile- or adult-biased removal, demonstrating the potential shifts in social structure as age structure is altered. The left column shows three initial social networks of 50 individuals with an equal (top), juvenile-biased (middle) and adult-biased (bottom) age distribution. Adults are shown in blue, subadults in green and juveniles in yellow. In these networks, we assume that the tendency to socialize decreases with age, i.e. juveniles are about six times more likely to socialize than adults. Underneath each social network, we present the network density (the number of existing connections divided by all possible connections), which gives a measure of how well individuals are connected. The central and right columns illustrate the hypothetical changes in network structure following juvenile-biased (central) or adult-biased (right) removal, i.e. under the juvenile-biased removal, juveniles had an 80% chance of being removed compared to adults and subadults (10% chance of removal each). In each case, 10 individuals were removed. Such effects of age distribution on social network structure should be assessed using empirical data from wild populations (see recent work [58,274]).

Recent advances have established social network analysis (SNA) as an increasingly powerful tool for understanding the causes and consequences of sociality in a range of evolutionary and ecological contexts [266–270]. By using SNA, individuals are studied as ‘nodes’ in a network, that are connected by ‘edges’ defined by social interactions [62,271,272]. Through this, the diverse range of associations between individuals are quantitatively assessed, such that hypotheses on the patterning of social processes and overall social structure can be tested in a generalized manner, providing insight into population-level behaviour. This allows examination of how individuals affect social processes and the emergent sociality of a group, such as the social transmission of behaviour, information or disease. Furthermore, including individual-level phenotypes (such as sex, size, etc.) in SNA allows for the quantitative link between such phenotypes, their associated social network metrics and group-level sociality. Although age itself is not a phenotype but rather represents a temporal parameter, it is associated with biological variance in various individual-level phenotypes and has a quantitative value which can be used in SNA. Specifically, due to the previously discussed effects of age on individual sociality, it is likely that age structure will influence interactions and relationships, thus necessarily shaping the overall social network and processes operating within it [58,273–276] (figure 2). For example, recent work by Siracusa *et al*. [58] assesses how changes in social behaviour in free-roaming rhesus macaques affect emergent social structure using SNA on empirical data. The results revealed that ageing female macaques became less indirectly connected for some, but not all, network measures. Further, the authors use agent-based models to understand the extent at which age-based social differences and certain age distributions would result in changes to the overall social network structure (similar to that presented in figure 2), but also reveal that variation in age structure does not relate to the structure of the network in this species. Such research is encouraging in that it shows the applicability of SNA in uncovering links between age, individual social behaviour and overall social structure.

Here, we suggest the wider use of SNA to study how age influences societies through three main routes. First, there are many detailed social networks that have been collected across numerous animal populations globally, which could be collated to test for relationships between age, social interactions and the emergent social structure. Second, by combining datasets that describe life-history attributes within animal populations [277,278] with their associated network datasets, it can be established how key demographic factors (such as birth and death rates) interact with individual ageing to affect how societies change with time. Finally, simulation modelling techniques could be applied to empirical data to assess how selection for particular age-related phenotypes, together with trans-generational processes such as inheritance and vertical transmission, shape inter-generational social structure.

Furthermore, an advantage of non-human animal populations is that they present several options to experimentally manipulate individual social behaviour, the social network or age structure to test proposed hypotheses using SNA. For instance, previous social network studies in wild great tits have used experimental removals to examine the effects of the loss of conspecifics on social behaviour and network structure [127] and used automated selective feeding stations to apply individual-level treatments to manipulate social structure. This has allowed researchers to experimentally impose social segregation of groups [279], alter the pathways of social information flow [280], assign foraging locations based on individuals’ age [281] and manipulate individuals’ social centrality [282]. In the future, such manipulations could be used to specifically manipulate local age structure and examine the direct consequences for social behaviour and arising social processes, which has recently been achieved using captive populations of forked fungus beetle (*Bolitotherus cornutus* [274,275]. Conversely, manipulations could be used to alter wild populations’ social structure and assess the impact this has on group demographic rates and resulting age structure, which similarly was recently explored using experimental populations of forked fungus beetle to evaluate multilevel selection as variation in group network structure interacts with demographic rates [283].

The use of SNA to study the effects of age structure in wild populations begs the question of how best to quantify this demographic property. In the literature, age structure is often used as a qualitative term, with little emphasis on how to examine it quantitatively. This may be because it is challenging for a scalar index to convey all information contained in a vector—in this case the relative composition of individuals in every age-cohort [70]. This differs from many other demographic characteristics that can be captured in a single statistic, such as population size, growth rate or sex ratio. Typically, animal population age structure is quantified as either the mean or median age of a population [70,284–286], or as the proportion in a given age-cohort, such as prime-aged or juvenile individuals [65,70,99,208,209,265,284,287–292]. While these statistics contain information regarding the central tendency and aspects of skew, we suggest future research should re-establish quantitative definitions of age structure such that maximum information on the distribution of age can be captured, upon which hypotheses can then be tested. This could be done through greater application of research from human population ageing [86,88,293,294]. For example, the *aged-child ratio* is the ratio of the number of elderly persons to the number of children, thus considering both ends of the age structure simultaneously. It is represented by the formula

,$$\frac{P^{65+}}{P^{0–14}}100$$

where $P^{65+}$ is the proportion of over 65-year-olds in the population and $P^{0–14}$ is the proportion of children 0 to 14 years old [293]. Adapting the aged-child ratio may be a useful way of quantifying animal population age structure, for example, by substituting the proportion of 65+-year-olds with the proportion of senescent individuals, and the proportion of 0- to 14-year-olds with the proportion of juveniles or sexually immature individuals.

In addition to suggesting the application of human ageing studies to inspire quantitative definitions of age structure, we also identify that explicit methodological studies can be used to define quantitative measures of ecological and evolutionary mechanisms or characteristics. For example, much research has been devoted to developing quantitative definitions of reproductive skew in populations, such that it can be studied in statistical terms with greater biological relevance [295–297]. We, therefore, suggest that future research should endeavour to determine new mathematical estimations of animal population age structure. This would improve studies of age structure and sociality by optimizing the amount of information on the distribution of age across a population, allowing the incorporation of age structure in the use of statistical approaches (such as SNA) and permitting direct comparison of age structure and related processes between populations, even of different species.

### (b) Social contagions in relation to age structure

Age structure is expected to affect how information, behaviours and diseases spread through populations by influencing social connections between individuals. Of these, the transmission of disease has received the most attention. For example, morbidity and mortality in wild bird influenza outbreaks are age-specific, where the youngest mute swans (*Cygnus olor*) die 16.8 times more frequently than birds of other ages [298–300]. As a result of this age-specificity in infection, individuals of separate ages differ in their likelihood of transmitting disease [301–303]. Such effects may be exacerbated by social structure, because of age-related variation in social association [304–307]. However, age structure may also influence the transmission of information or behaviours, as well as disease. This may not be apparent if considered as a ‘simple contagion’, whereby the likelihood of learning is assumed to be determined by the total number of network connections to informed individuals [77,308–310]. However, instead, age-specificity in social learning means that behaviours may spread as ‘complex contagions’, whereby transmission is not only determined by the number of connections but also by specific rules governed by age that affect uptake of the behaviour [77,78]. Thus, when considering complex patterns of transmission through SNA, age effects on social contagions might be detected.

Such age effects exist because the age composition of dyads that make up groups influences whether an individual learns from another, and how quickly information is transmitted [311–316]. Furthermore, the age of individuals in such dyads will affect how long behavioural change will persist [317], influencing the likelihood that a behaviour will continue to spread through a population. This is caused by age-specific abilities to acquire, process, utilize and transmit information [79,318]. On a population level, this means that age structure might influence if and how quickly behaviour spreads, dependent on the probability of transmission between different age classes, or due to critical periods in development where social learning is easier [76]. For example, in troops of Japanese macaques (*Macaca fuscata*) with missing age classes (and therefore an abnormal age structure), stone-handling behaviours are less likely to spread and are performed less frequently [319]. Similarly, when novel or invented behaviours are restricted to one age class, they may be less likely to spread or be maintained within a population [320,321]. The causal effects of age on social transmission of behaviour should receive more attention and is an example of how SNA could be used to assess the effects of age structure on sociality.

### (c) Human impact on wild populations’ social ageing

Generating a better understanding of the link between age structure and social behaviour is crucial because human activities are increasingly modifying wild population demographics [292,322–326]. Human-induced environmental changes are diverse, ranging from structural modifications to the physical environment, such as landscape fragmentation, pollution and anthropogenic food subsidies [327–329], to changes in the social environment by influencing population size, composition and social interactions [330,331]. Importantly, changes in animal sociality can be mediated by human-induced changes in population age structure. Here, we briefly review two human activities— supplemental feeding and the selective harvesting of wild animals—and their potential impact on population age structure and sociality.

Supplemental feeding, such as bird feeding stations, can affect age structure by artificially increasing survival rates in certain age-cohorts [332,333]. For example, adult tit species (Paridae) often have higher winter survival than yearlings, presumably because of more foraging experience and higher dominance [334,335]. Supplemental feeding increases the survival of yearlings [334] and may thus lead to a bias in population age structure towards younger age classes. Furthermore, food supplies can impact age structure if age classes respond differently to anthropogenic food. For example, the provisioning of food is often used in the conservation of scavenger populations such as the bearded vulture (*Gypaetus barbatus*). Contrary to expectation, anthropogenic feeding sites have been found to increase the survival of sub-adults but not adults in this species, presumably because adult birds foraged less frequently on these food types, leading to on average younger populations [336]. By increasing the survival of younger cohorts, supplemental feeding thus has the potential to drive changes in emergent social structure and functioning by promoting social processes that are performed to a greater extent in younger age cohorts.

One of the best documented cases of human activities impacting wild populations’ age structure is selective harvesting. Hunting and fishing often target individuals with specific phenotypic traits [337–341]. Unsustainable trophy hunting selects individuals with the most attractive ornamental traits such as horns, antlers, plumage and body size, which often correlates with age, thus often leading to age-specific removal of individuals [339]. For example, human hunters select on average younger female elks (6.5 years) with greater reproductive value compared to those selected by natural grey wolf (*Canis lupus*) predators (13.9 years). Therefore, by primarily removing prime-aged females, humans may have a strong impact on the future population viability and the emergent age structure of elks [342]. Age-specific harvesting is particularly evident in fish populations, where larger and older fish which contribute disproportionately to spawning and population growth are often the same cohort which are removed the most through commercial harvesting, thus causing truncations in the age structure and damaging the future resilience of populations [343–350]. Related, illegal wildlife trade can result in age-biased removal of individuals [351,352]. For instance, poaching of various parrot species (order Psittaciformes) is biased towards the extraction of fledglings because they are easier to locate and catch than adult birds [352]. Hence, in addition to decreases in population size, certain harvesting practices can alter population age structure, which may have consequences for population social structure and functioning (for example, see effects of juvenile-biased removal on network density in figure 2).

### (d) Advancing our understanding of social senescence

Finally, we briefly highlight the importance of advancing our understanding of social senescence. In this review, we have considered social ageing as a process of general age-related changes in social behaviour as individuals progress through time, and have discussed patterns that are likely to emerge in population-level sociality given variation in age structure. We hope this may also provide an initial base from which further research can assess and build a cross-species understanding of social senescence specifically.

**Figure 3.**
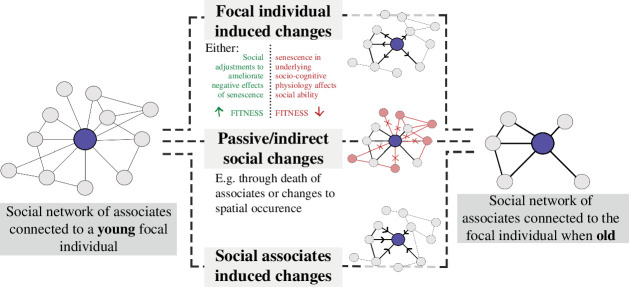
Different mechanisms that could result in the same late-life changes in social selectivity with age. In each network, the focal individual is represented by the dark blue node.

Senescence is the decline in organismal functioning with old age and thus is associated with decreased fitness as selection is weakened in late life [6–13]. Such senescence is evidenced in wild populations, with old-age-related changes in survival probability, reproduction and other, typically physiological, traits [18]. However, while physiological senescence is evident, our understanding of social senescence remains considerably less clear. Specifically, while age-related changes in social behaviour occur with old age, the process behind such changes is ambiguous. Indeed, there is currently limited knowledge on whether age-related changes in social behaviour are generally as a result of senescence (i.e. declining physiological health) or other mechanisms, and whether old-age-related changes in social behaviour hold negative outcomes for the organism. For example, changes in social selectivity with age (where older individuals have fewer but stronger relationships, as discussed previously) could be generated by several different mechanisms while producing similar patterns, and may have positive or negative effects (figure 3). First, late-life-related social change might be induced by the focal individual, but this could either be associated with increasing fitness if they are adjustments in social behaviour to ameliorate the negative effects of senescence or decreasing fitness if mediated by senescence in underlying socio-cognitive physiology. Second, old age social change may be unrelated to active changes in social behaviour but instead be a result of other processes with old age, such as changes in spatial occurrence or death of conspecifics. Finally, social traits are influenced not only by genes carried by focal individuals (direct genetic effects) but also by social partners (indirect genetic effects) as dyadic relationships are as a result of more than one individual [96,268,353–355]. Therefore, late-life social change might be primarily mediated by changes in the social behaviour of associates. Work has begun to assess the role of social senescence in driving late-life changes in social behaviour versus other mechanisms [34,48,356], along with the consequences of this for individuals’ fitness, but more research is needed to gain a generalized understanding of social senescence and its role in natural populations.

## 4. Concluding remarks

We have highlighted the roles that population age structure and sociality each play in influencing variation in the other. However, the relationship between these variables remains little studied in the wild. We have further highlighted the opportunities to be gained by using SNA in combination with data from natural populations, and we hope that this inspires future research that uses SNA to examine the causal links between variation in age structure and the social functioning of wild populations. Understanding the consequences of variation in age structure on population-level processes is timely, given the increasing impact of anthropogenic activity on population age structure, both indirectly as environmental change impacts the demography and emergent age structure of populations and directly as age structure is altered through hunting and harvesting. Furthermore, human populations are rapidly ageing for the first time in history. Through advancements in our understanding of age structure in natural populations, greater insights into whether there are fundamental rules of how societies age and the potential social implications of this across systems may be possible. Our hope is that future research will provide new understanding of how age shapes social behaviour and emerging societal structure, the ecological and evolutionary forces that mediate these effects, and the consequences in turn of variation in age structure for fundamental social processes.

## Data Availability

This article has no additional data.
